# Metabolic profiling reveals interleukin-17A monoclonal antibody treatment ameliorate lipids metabolism with the potentiality to reduce cardiovascular risk in psoriasis patients

**DOI:** 10.1186/s12944-021-01441-9

**Published:** 2021-02-18

**Authors:** Han Cao, Shengmin Su, Qi Yang, Yunchen Le, Lihong Chen, Mengyan Hu, Xiaoyu Guo, Jie Zheng, Xia Li, Yunqiu Yu

**Affiliations:** 1 School of Pharmacy, Fudan University, Shanghai, 201203 People’s Republic of China; 2 Department of Dermatology, Ruijin Hospital, School of Medicine, Shanghai Jiaotong University, Shanghai, 200025 People’s Republic of China

**Keywords:** Psoriasis, Cardiovascular diseases, Ixekizumab, Lipids, Metabolism, IL-17A monoclonal antibody, Lysophospholipids

## Abstract

**Background:**

Psoriasis is a common chronic inflammatory skin disease associated with overproduction of interleukin-17A (IL-17A). IL-17A monoclonal antibodies (mAbs) have shown clinical efficacy in psoriasis patients. Although a series of different overlapping mechanisms have been found to establish a link between psoriasis and cardiovascular diseases, the underlying mechanisms of the two types of diseases and the potential efficacy of IL-17A mAbs in amelioration of cardiovascular comorbidities remain unclear.

**Methods:**

Serum samples from two study cohorts including 117 individuals were analyzed using a high-throughput UHPLC-MS platform. Non-targeted metabolic profiling analysis was first conducted with samples from 28 healthy individuals and from 28 psoriasis patients before and after 12-weeks of ixekizumab treatment in study cohort 1. Study cohort 2 was additionally recruited to validate the correlations of the identified metabolites with cardiovascular diseases.

**Results:**

A total of 43 differential metabolites, including lysophospholipids, free fatty acids, acylcarnitines and dicarboxylic acids, were accurately identified in study cohort 1, and the analysis showed that lipid metabolism was impaired in psoriasis patients. Compared with healthy individuals, psoriasis patients had higher levels of lysophosphatidylcholines, lysophosphatidylinositols, lysophosphatidic acids and free fatty acids, but lower levels of acylcarnitines and dicarboxylic acids. The identified dicarboxylic acid levels were inversely correlated with psoriasis area and severity index (PASI) scores (*P* < 0.05). The results for study cohort 2 were largely consistent with the results for study cohort 1. Moreover, the levels of all identified lysophosphatidylcholines were higher in psoriasis patients with coronary heart diseases than in psoriasis without coronary heart disease. Notably, most of these lipidic changes were ameliorated by ixekizumab treatment.

**Conclusion:**

The results of this non-targeted metabolomic analysis indicate that treatment with IL-17A mAbs can not only ameliorate psoriasis lesions but also restore dysregulated lipid metabolism to normal levels in psoriasis patients. Considering that dysregulated lipid metabolism has been regarded as the critical factor in cardiovascular diseases, the recovery of lipid metabolites in psoriasis patients indicates that IL-17A mAbs might have the potential protective effects against cardiovascular comorbidities.

**Supplementary Information:**

The online version contains supplementary material available at 10.1186/s12944-021-01441-9.

## Introduction

Psoriasis, one of the most common chronic inflammatory skin diseases, features metabolic and cardiovascular comorbidities [1]. The association of cardiovascular diseases (CVDs) with psoriasis was first observed in the 1890s [2]. An increased risk of CVDs in psoriasis patients has since been reported by a number of studies [3–5]; thus, psoriasis is an additional risk factor aside from traditional cardiovascular risk factors. Compared with the non-psoriatic population, psoriasis patients have a higher prevalence of types 2 diabetes mellitus (13.9% vs 7.4%), dyslipidaemia (28.8% vs 17.4%) and arterial hypertension (31.2% vs 19.0%) [6]. In addition to the abnormal immune cell responses observed in the pathogenesis of psoriasis, recent pathophysiological research has focused on activation of the interleukin (IL)-23/IL-17 axis, which enhances abnormal keratinocyte proliferation and induces psoriasis [7]. Although the exact role of IL-17A in CVDs is still debatable, aggregated IL-17- producing cells and enhanced IL-17A levels have been observed in atherosclerotic lesions [8, 9]. The “two plaques, one syndrome” hypothesis was proposed since the molecular mechanisms of these two diseases bear a remarkable resemblance to T cell- mediated inflammation [10]. Although the increased risk of CVDs in psoriasis patients may partly be explained by the hypothesis that chronic skin inflammation and concomitant proinflammatory cytokine activity promote the development of CVDs, the underlying mechanisms remain unclear [11]. Ixekizumab, a recombinant humanized IgG4-κ monoclonal antibody (mAb) that selectively binds and neutralizes IL-17A, has been employed clinically for psoriasis since 2015 [12]. The correlation between the two types of diseases and its effect on cardiovascular comorbidities has aroused great concerns.

Metabolomics, an important member of the omics field, can be used to elucidate the complex interactions between individual genetic inheritance and constantly changing environments via identification and measurement of small-molecule metabolites [13]. However, published metabolomic analyses related to psoriasis or CVDs have focused mainly on pathophysiologic mechanisms between patients and healthy people [14, 15]. In addition to playing important roles in CVDs, lipids also have critical functions in skin health, and abnormal lipid metabolism is involved in the pathogeneses of common skin diseases [16, 17]. Lipidomics has been comprehensively applied to describe the relationships between lipids and dermatologic diseases, especially those regarding the structure and function of the end-products of lipid metabolism [18, 19]. Several biologic agents have been successfully used in the treatment of psoriasis and have an acceptable safety and tolerability, but few studies profiled altered metabolites, especially lipids, before and after biologic agent treatment in psoriasis patients.

Considering the remarkable resemblance of the pathogeneses in psoriasis and CVDs, in this study, non-targeted metabolomics based on a high-throughput ultra-performance liquid chromatography mass spectrometry (UHPLC-MS) platform was used to clarify the metabolic alterations in IL-17A mAb-treated psoriasis patients in order to provide insight into the potential convergent mechanism in psoriasis and CVDs.

## Methods

### Study design

**Fig. 1:**
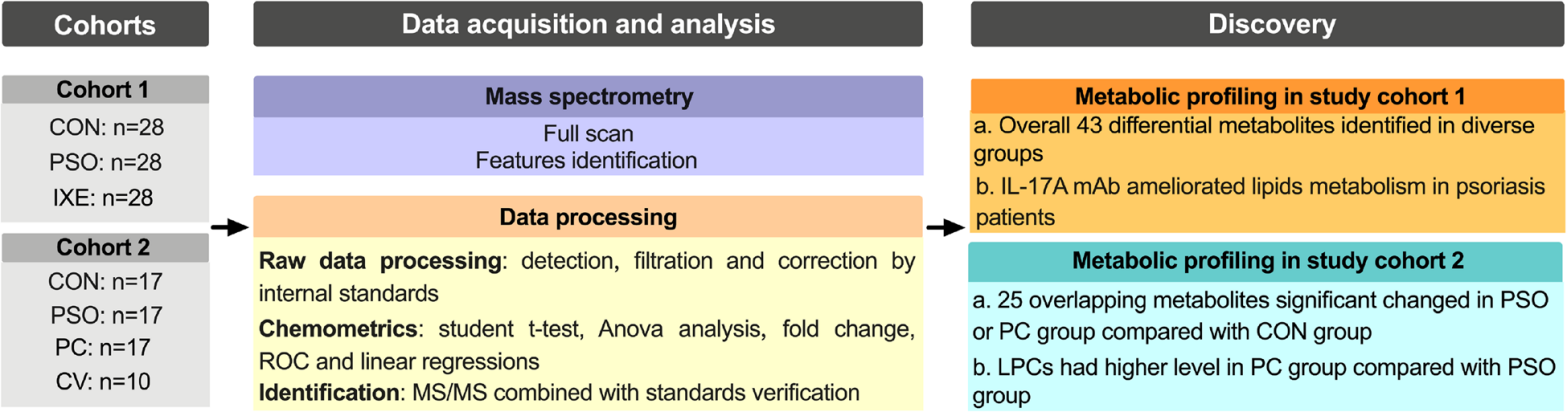
Workflow of this study. CON: group of healthy controls; PSO: group of psoriasis patients; IXE: group of ixekizumab-treated psoriasis patients; PC: group of psoriasis patients with coronary heart disease; CV: group of coronary heart disease patients without psoriasis

This study included two study cohorts, and the protocol was reviewed and approved by the Ethics Committee of Ruijin Hospital of Shanghai Jiao Tong University. Written informed consent was obtained from each patient and healthy individual before enrolment. This study included all the participants in the two study cohorts recruited from Ruijin Hospital (Table 1). In the two study cohorts, none of the healthy individuals had diabetes, hypertension, hyperlipidaemia or other obesity-related metabolic diseases. None of the patients were prescribed anti-inflammatory drugs or other anti-inflammatory treatments for approximately 4 weeks. Psoriasis severity was assessed via psoriasis area severity index (PASI) scoring, which combines evaluation of erythema, induration, and desquamation within each lesion [20]. Healthy individuals matched by age and gender were collected from a physical examination centre centre of Ruijin Hospital of Shanghai Jiao Tong University. In study cohort 1, serum samples were collected from 28 healthy people (the CON group) and 28 psoriasis patients treated with ixekizumab at baseline (the PSO group) and 12 weeks after treatment (the IXE group). The 28 psoriasis patients in the study cohort 1 and 6 psoriasis patients with coronary heart disease in the study cohort 2 received subcutaneous injections of 80 mg of ixekizumab every 2 weeks or every 4 weeks after a starting dose of 160 mg [21–23]. Metabolic profiling analysis was first conducted in the CON, PSO and IXE groups of study cohort 1. To validate the correlations of the identified differential metabolites and CVDs, the second study cohort was recruited with additional groups of psoriasis patients with coronary heart disease (the PC group) and coronary heart disease patients without psoriasis (the CV group) (Fig. 1). The psoriasis patients in the second study cohort were deemed to have coronary heart disease as a complication if coronary computed tomography angiography revealed one or more atherosclerotic lesions with moderate (50 to 70%) or severe (> 70%) lumen stenosis. Considering that psoriasis patients with cardiovascular comorbidities were mainly elderly and that age is the critical factor in CVDs, additional healthy people (the CON group) and psoriasis patients (the PSO group) of comparable age were also recruited for study cohort 2. For serum collection, blood samples were collected in EDTA tubes, centrifuged for 5 min at 3000 rpm within 4 h to collect serum and immediately frozen at − 80 °C until processing.

**Table 1:** Demographic information on the two study cohorts

	Cohort 1			Cohort 2			
	CON (*n* = 28)	PSO (*n* = 28)	IXE (*n* = 28)	CON (*n* = 17)	PSO (*n* = 17)	PC (*n* = 17)	CV (*n* = 10)
Male/Female	19/9	19/9	19/9	13/4	15/2	14/3	8/2
Age (years)	45 ± 11.6	45 ± 11.5	45 ± 11.5	62 ± 6.6	60 ± 7.1	63 ± 7.3	68 ± 14.3
BMI	24.1 ± 2.8	24.9 ± 2.8	26.1 ± 3.1	23.9 ± 2.4	24.1 ± 3.1	24.9 ± 3.9	23.3 ± 2.4
PASI	n/a	24.4 ± 8.7	0.4 ± 0.3	n/a	21.70 ± 12.2	16.87 ± 15.99	n/a

Values are reported as mean ± SD

*BMI* body mass index, *PASI* psoriasis area and severity index, *CON* group of healthy controls, *PSO* group of psoriasis patients, *IXE* group of ixekizumab-treated psoriasis patients, *PC* group of psoriasis patients with coronary heart disease, *CV* group of coronary heart disease patients without psoriasis

### Mass spectrometry

A total of 120 μL of cold methanol containing internal standards was mixed with 30 μL of serum. The mixture was vortexed for 5 min and then kept at room temperature for 10 min to allow protein precipitation. Hexadecylamine and tridecanoic acid (Sigma-Aldrich, MO, USA) were used as internal standards in positive mode and negative mode, respectively. After centrifugation at 12000 rpm for 5 min, the supernatant was collected for UHPLC-MS analysis. Quality control samples (QCs) were obtained by mixing 20 μL from each serum sample.

UHPLC-MS analysis was conducted on a 1290 Infinity UHPLC system coupled to a 6530 iFunnel ESI-Q-TOF mass spectrometer (Agilent Technologies, CA, USA) that was equipped with a degasser, binary pump and thermostatically controlled autosampler. Chromatographic separation was carried out on an ACQUITY UPLC HSS T3 column (2.1 mm × 100 mm, 1.8 μm, Milford, MA, USA) with 0.1% formic acid in either water (A) or acetonitrile (B) as the mobile phase [24, 25]. The percentage of mobile phase A was kept at 99% for the first 1 min and decreased linearly to 60%, 50% and 35% over the next 4 min, 3 min and 8 min, respectively, under a flow rate of 0.3 mL/min. From 8 to 16 min, the percentage of mobile phase A was further decreased to 24% before being decreased to 0% and maintained for 5 min. Ten microlitres of each sample was injected, and the column was held at a constant temperature of 35 °C. The QCs were analyzed at regular intervals throughout the whole analytical run.

Real-time mass calibration was carried out by monitoring two reference compounds each in positive mode (*mz* 121.0509 and *m/z* 922.0098) and negative mode (*m/z* 112.9856 and *m/z* 1033.9881). Acquisition was carried out at a resolution of 32,000 in centroid mode with one spectrum per second in the 50–1050 *m/z* range. The electrospray ionization (ESI) source parameters were set as follows: desolvation gas, nitrogen at 10 L/min; nebulizer pressure, 40 psi; fragmentor voltage, 175 V; capillary voltage, 3500 V; and gas temperature, 350 °C.

### Data analysis

Raw data were acquired with a MassHunter workstation and converted into mzData format with MassHunter Qualitative Analysis software (B.06.00). Further data processing steps were conducted at XCMS-Online (https://xcmsonline.scripps.edu), including feature detection, peak alignment and retention time correction. The intensity of each feature was corrected by the response of the internal standard in the same sample before statistical analysis. The processed data were subjected to principal components analysis (PCA) and orthogonal partial least squares discrimination analysis (OPLS-DA) after raw data filtering and processing.

The metabolites were identified performed according to rules set out by the Chemical Analysis Working Group of the Metabolite Standards Initiative [26]. The criteria for feature selection were set as a *P*-value < 0.05 from t-test analysis and a variable importance in projection (VIP) score > 1 from OPLS-DA. The VIP score of a metabolite, which is calculated as a weighted sum of the squared correlations between this metabolite and the derived OPLS-DA components, can be used to measure the importance of this metabolite in the multivariate analysis [27, 28]. In the current study, identified metabolites were labelled as level 1 if confirmed with reference standards or as level 2 if the MS/MS spectra matched with those from the Human Metabolome Database (www.hmdb.ca) when reference standards were not available [29]. MS/MS analysis was also conducted on the same mass spectrometer. In MS/MS analysis, three collision energies (10 eV, 20 eV and 40 eV) were used on an additional scan following the precursor ion full scan. To standardize the comparison, all the serum samples and reference standards were subjected to the same MS/MS method, and the same injection volumes were used.

### Chemometrics

The raw data were logarithmically transformed and tested for normality before the means were compared between different groups. If normality was assumed, Student’s t-test was applied; otherwise, a nonparametric method was used. In study cohort 1, the differences in means between the CON and PSO groups were analyzed by Student’s t-test if the assumption of normality was met. For the PSO group and the IXE group, a paired t-test was applied when the normality assumption was met; otherwise, the Wilcoxon method was used. In study cohort 2, ANOVA was performed to compare the means among the four groups, and Dunnett’s t-test was used for post hoc comparisons against the CON group. The means of the PC group and PSO group were compared with independent t-tests if normality was met. For the six individuals who received ixekizumab treatment in the PC group, a paired t-test was applied when the normality assumption was met; otherwise, the Wilcoxon method was used. All of the abovementioned statistical analyses were performed in SPSS v20 (IBM, IL, USA). Linear regression models, prediction plots and receiver operating characteristic (ROC) curves were generated using Prism 8.0 (GraphPad Software Inc., San Diego, CA, USA). To visualize the differentiation between different groups, PCA, sparse partial least squares discriminant analysis (sPLS-DA) and OPLS-DA were performed using MetaboAnalyst 4.0 (http://www.metaboanalyst.ca/).

## Results

### Non-targeted metabolomics reveals the metabolic profiles of psoriasis patients before and after ixekizumab treatment

Whether in positive ion or negative ion mode, the IXE group was invariably between the CON and PSO groups, which indicated that ixekizumab treatment shifted the metabolic profiles of psoriasis patients toward a normal status (Fig. S1a, b). To better investigate the abnormal metabolites causing the different metabolic profiles, OPLS-DA was used for subsequent PSO/CON and IXE/PSO paired comparisons (Fig. S1c-f).

**Fig. 2:**
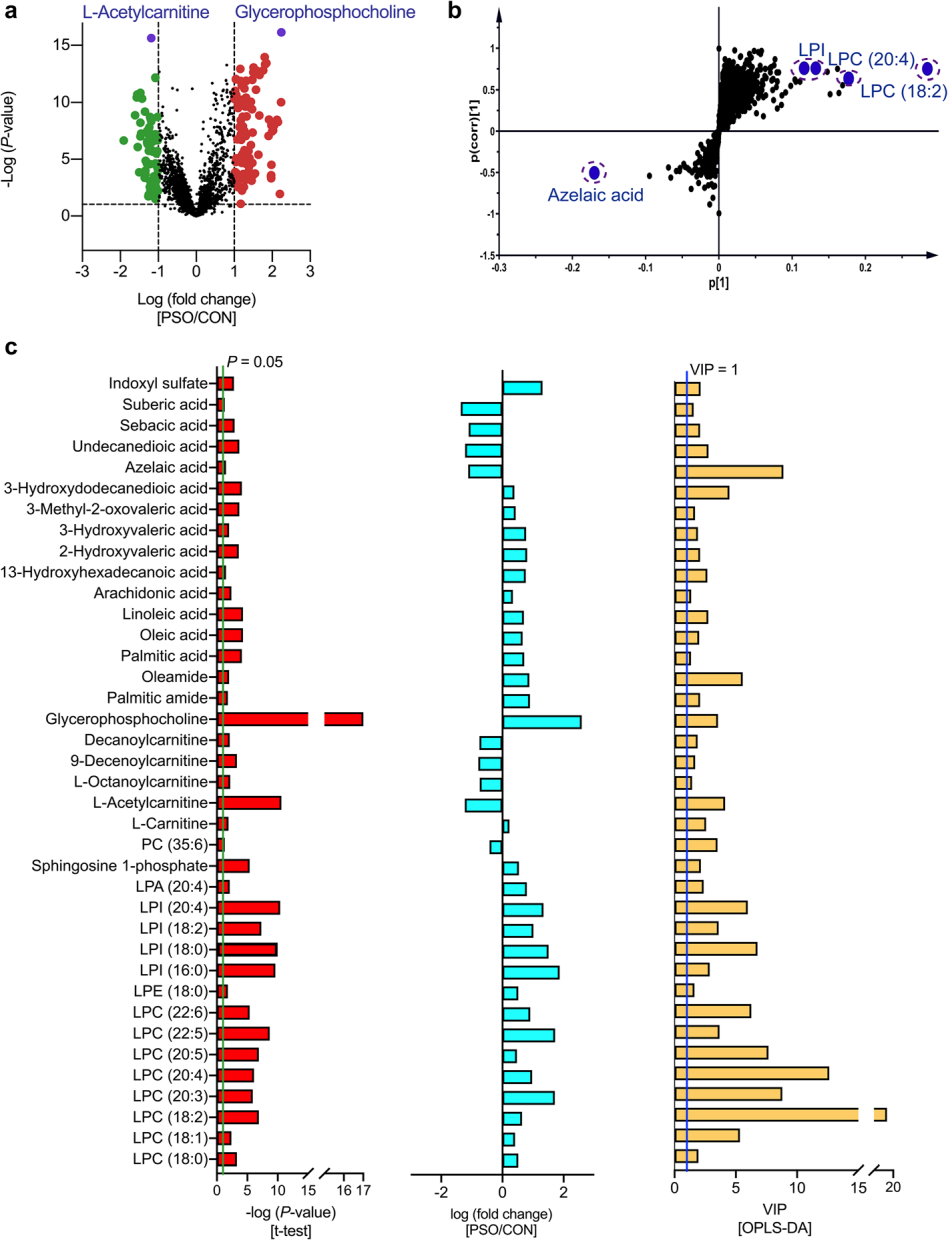
Screening criteria for the differential metabolites identified in the PSO/CON comparison in study cohort 1. **a.** Volcano plot showing the variations in metabolites in the PSO/CON comparison according to the -log(*P*-value). **b.** S-plots for covariance and reliability correlations from OPLS-DA in the PSO/CON comparison. **c.** Thirty-seven identified differential metabolites in the PSO/CON comparison. The Bar plots represent, from left to right, the −log(*P*-value) outcomes from the t-test, the fold changes and the VIP values obtained from OPLS-DA. CON: group of healthy controls; PSO: group of psoriasis patients

The constructed multivariate models, in conjunction with univariate statistical analysis, revealed 37 differential metabolites in the PSO/CON comparison (Fig. 2), and 31 differential metabolites were accurately identified in the IXE/PSO comparison (Fig. S2). In total, 43 metabolites contributed most to the differentiation of the groups in study cohort 1, including lysophospholipids (LPLs), free fatty acids (FFAs), dicarboxylic acids (DAs) and acylcarnitines (Table S1).

ROC curve analysis is generally considered to be the gold standard for assessment of biomarker performance. The area under the curve (AUC) values of the ROC curves of the 37 differential metabolites in the PSO/CON comparison were higher than 0.7, indicating that the metabolites can be regarded as potential biomarkers for psoriasis (Fig. S3) (Table S2). Notably, the AUC values of all identified LPLs except lysophosphatidic acid (LPA) (20:4) were higher than 0.7 in both the PSO/CON comparison and the IXE/PSO comparison, which indicated that these identified LPLs may serve as predictive markers of the efficacy of IL-17A mAb treatment in psoriasis (Fig. S3) (Table S2).

### IL-17A mAb treatment ameliorated dysregulated lipid metabolism in psoriasis patients

**Fig. 3:**
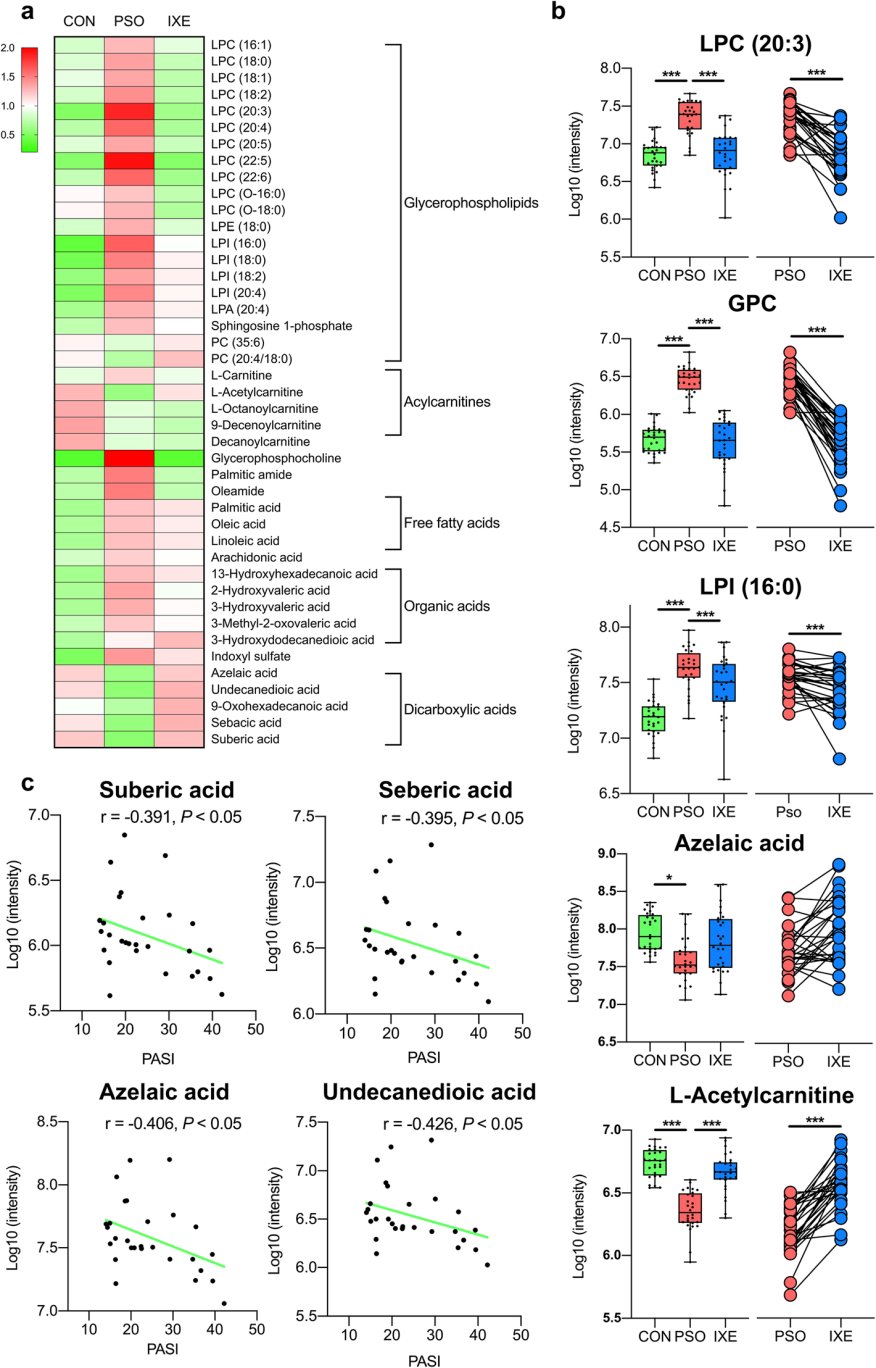
IL-17A mAb ameliorates dysregulated lipid metabolism in psoriasis patients. **a.** Heatmap of the 43 identified differential metabolites in the study cohort 1. The color represents the average normalized intensity of each metabolite. **b.** Box plots of highlighted metabolites in study cohort 1. *P < 0.05, **P < 0.01, ***P < 0.001. **c.** Correlation analysis between the identified DAs and PASI scores of psoriasis patients in study cohort 1. CON: group of healthy controls; PSO: group of psoriasis patients; IXE: group of ixekizumab-treated psoriasis patients; PASI: psoriasis area and severity index

As visualized in the heat map, LPLs and FFAs were upregulated in psoriasis patients compared with healthy people, while DAs and acylcarnitines were altered in the opposite way (Fig. 3a). Lysophosphatidylcholines (LPCs), lysophosphatidylinositols (LPIs) and LPAs were significantly upregulated in the psoriasis patients. It has been demonstrated that circulating LPCs and LPAs have potent pro-inflammatory effects and are upregulated in the several inflammation-associated diseases, including psoriasis [30, 31]. Moreover, the presence of n-6 polyunsaturated fatty acids on LPCs strengthens the ability of LPCs to evoke an inflammatory response [32]. In this study, LPC (22:5) and LPC (20:3) with n-6 polyunsaturated fatty acids had the highest fold-change values among all the identified LPCs (Fig. 3b). In accordance with the increased levels of LPCs, the levels of glycerophosphocholine (GPC), a downstream product of LPCs, were also dramatically upregulated in psoriasis patients (Fig. 3b). As LPCs can be produced by phosphatidylcholines, the decreased levels of phosphatidylcholines further proved that the phospholipid pathway was disrupted. In addition to LPCs, members of another group of LPLs, LPIs, were also upregulated in psoriasis patients (Fig. 3b). Although LPIs are present at relatively lower concentrations in human blood than LPCs, they have abundant biological functions, including pro-inflammatory functions [33].

In Land’s cycle, FFAs can be produced from LPLs by phospholipase A [34]. As expected, FFA levels were higher in the serum of psoriasis patients than in that of healthy individuals. In contrast to FFAs, DAs and acylcarnitines exhibited lower levels in psoriasis patients than in healthy individuals (Fig. 3b). Since DAs are the intermediates in the ω-oxidation pathway and since acylcarnitines are the “vehicles” in the β-oxidation process, the decreases in the levels of these two metabolites indicated potential dysfunction of fatty acid decomposition that in turn resulted in elevations in the blood levels of FFAs. Accumulation of FFAs has been reported to constantly sensitize dendritic cells to amplify Th1/Th17 immune responses [35]; this mechanism was supported by the inverse correlation between DA levels and PASI scores (Fig. 3c).

After ixekizumab treatment, the most obvious metabolic changes in psoriasis patients were decreased LPCs and GPC levels. In particular, the levels of the aforementioned inflammation-associated LPC (20:3) and LPC (22:5) were drastically decreased in the treated patients. The PC levels increased synchronously with the decrease in LPC levels, and the PC and LPC levels both returned to normal. The downstream product of LPCs, GPC, showed the strongest decreasing trend among all the differential metabolites, which could be explained by the decreased in LPC levels. The changes in the average levels of acylcarnitines and FFAs were ameliorated at the same time, although the amelioration was not statistically significant. However, DAs were upregulated to normal levels in treated patients. These results indicate that treatment with IL-17A mAbs might not only ameliorate psoriasis lesions, but also restore the dysregulated lipid metabolism to normal levels in psoriasis patients.

### Common and specific metabolites in psoriasis patients with or without cardiovascular comorbidities

**Fig. 4:**
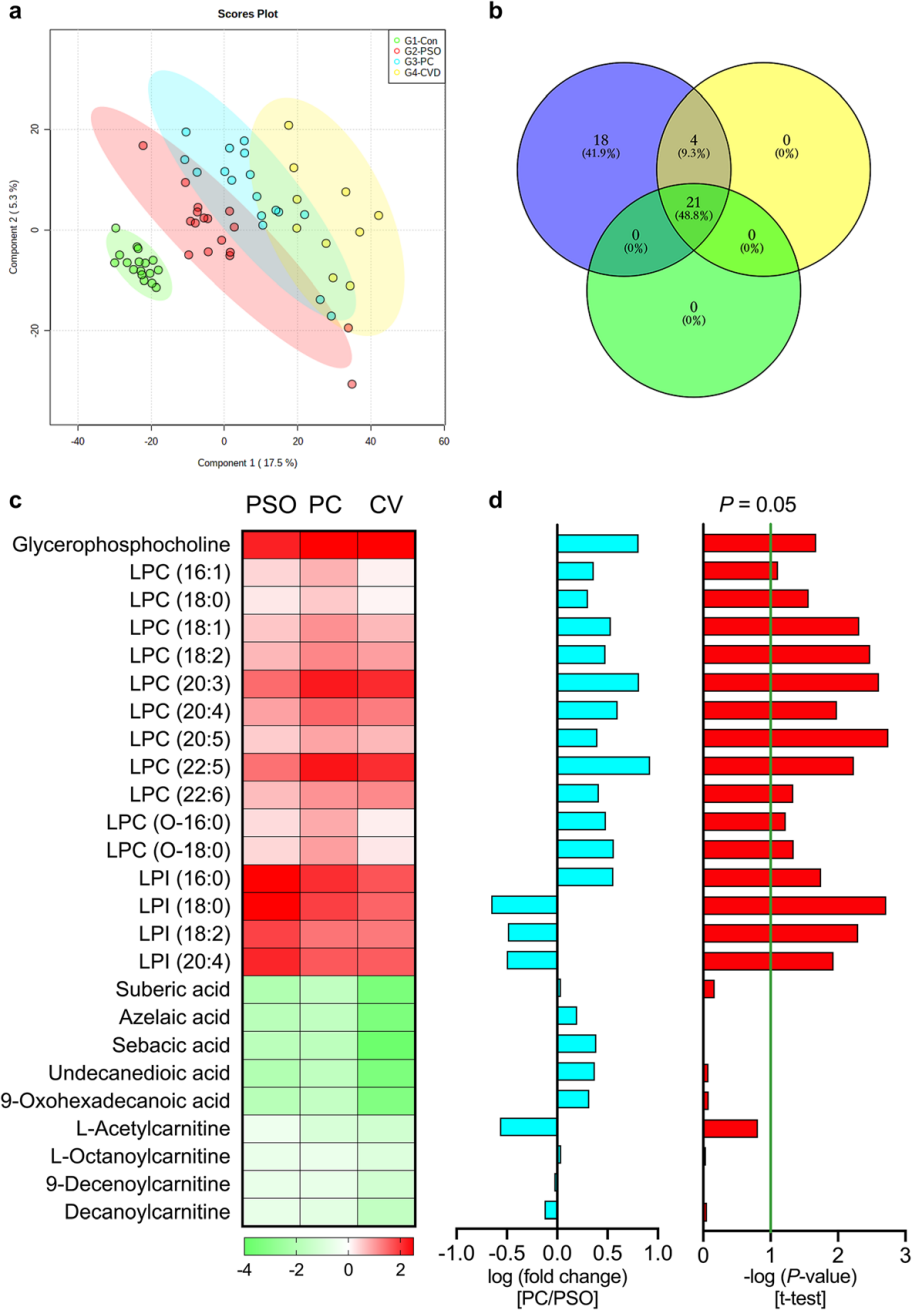
Common and specific metabolites in psoriasis patients with or without coronary heart disease. **a.** sPLS-DA score plots of the CON, PSO, PC and CV groups in study cohort 2. The CON group is indicated with green circles, the PSO group with red circles, the PC group with blue circles, and the CV group with yellow circles. **b.** Venn diagram of the 25 overlapping metabolites in the PSO, CV and PC groups compared with the CON group in study cohort 2. **c.** Heatmap of the 25 overlapping metabolites in the PSO, PC and CV groups (all compared with the CON group). **d.** The bar plots represent, from left to right, the log (fold change value) and -log(*P*-value) from t-test analysis in the PC/PSO comparison in study cohort 2. CON: group of healthy controls; PSO: group of psoriasis patients; PC: group of psoriasis patients with coronary heart disease; CV: group of coronary heart disease patients without psoriasis

The results of metabolomic analysis on the study cohort 2 largely conformed to the previous observations in study cohort 1. In sPLS-DA, the PC group was between the PSO and CV groups, and apparent separation was achieved among the four groups (Fig. 4a). Twenty-five of the differential metabolites identified in the PSO/CON comparison in study cohort 1 were demonstrated to also be significantly differentially abundant in the PSO, CV and PC groups compared to the CON group in study cohort 2 (Fig. 4b, c). Although all identified LPLs were upregulated in the PSO and PC groups of the two study cohorts, LPC levels were even higher in the PC group than in the PSO group (Fig. 4d). In study cohort 2, the aforementioned LPC (22:5), LPC (20:3) and GPC were not only drastically upregulated in the PSO group, but also upregulated in the PC group. Although all the identified LPIs were upregulated in the PSO groups of the two study cohorts, psoriasis patients with coronary heart disease had significantly lower levels than those without such diseases (Fig. 4d).

The trends of DA dysregulation in the patients of study cohort 2 were also consistent with the results for study cohort 1, but there were no significant differences between the PSO and PC groups (Fig. 4c, d). Considering that DA levels were inversely correlated with PASI scores in study cohort 1, this outcome was unexpected. The results of metabolic profiling for study cohort 1 demonstrated that the levels of acylcarnitines, which are critical carriers in fatty acid oxidation, were decreased in psoriasis patients. In study cohort 2, although the levels of medium-chain acylcarnitines were decreased in all patients, no significant differences were found between the PSO and PC groups; the results were consistent with the results obtained for DAs.

**Fig. 5:**
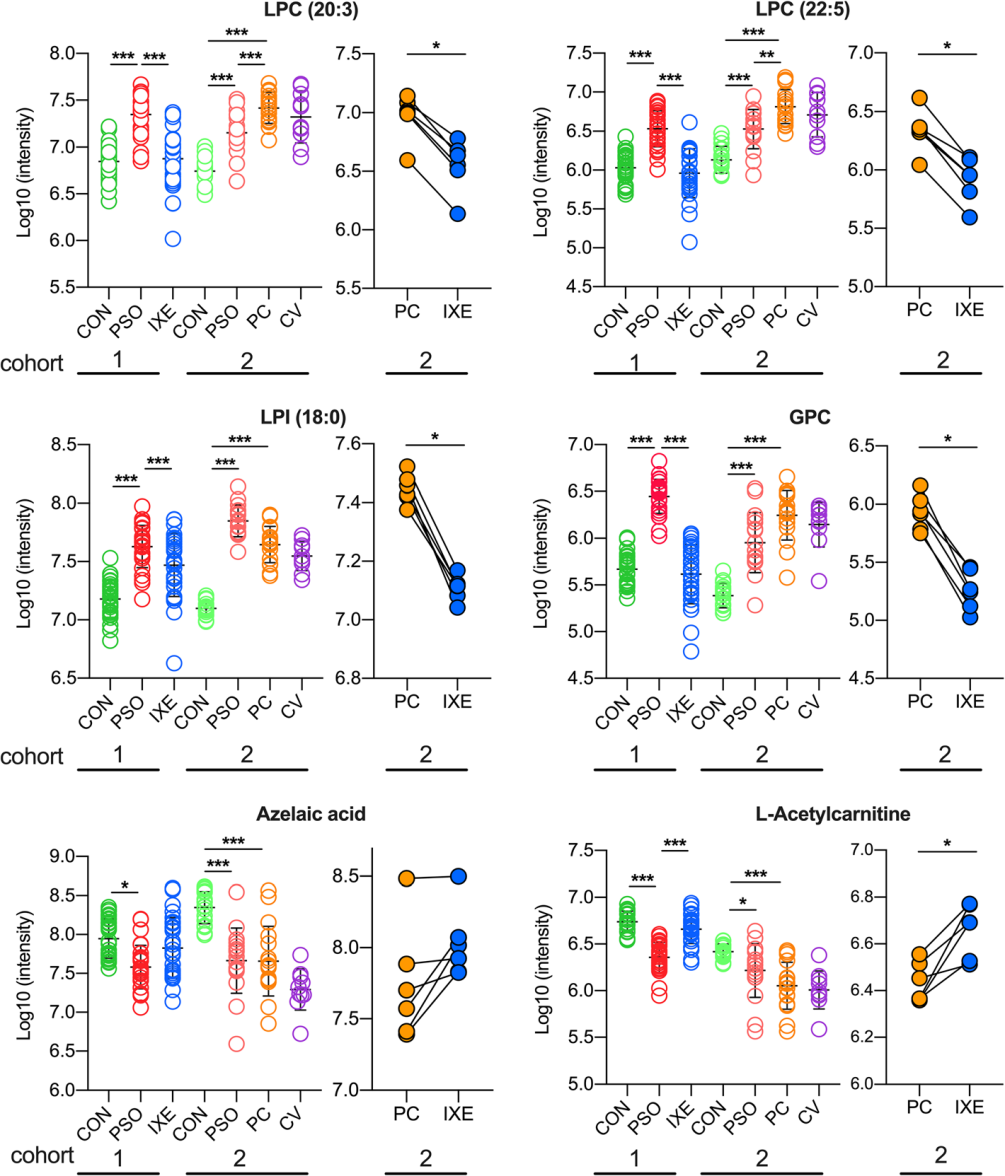
Scatter plots of highlighted metabolites in study cohorts 1 and 2. The metabolic changes in six ixekizumab-treated individuals from the PC group in the study cohort 2 conformed to previous observations in study cohort 1. *P < 0.05, **P < 0.01, ***P < 0.001. CON: group of healthy controls; PSO: group of psoriasis patients; IXE: group of ixekizumab-treated psoriasis patients; PC: group of psoriasis patients with coronary heart disease; CV: group of coronary heart disease patients without psoriasis

Although only six individuals in the PC group were treated with ixekizumab, the changes in lipid metabolism conformed to the previous observations in study cohort 1. Ixekizumab treatment restored LPLs, DAs and acylcarnitines to normal levels in psoriasis patients with coronary heart disease (Fig. 5). This result indicates that IL-17A mAbs might restore dysregulated lipid metabolism to normal levels in psoriasis patients with coronary heart disease.

## Discussion

In this study, metabolomic analysis was conducted on two study cohorts including healthy people, psoriasis patients before and after ixekizumab treatment, and psoriasis patients with coronary heart disease. In study cohort 1, the majority of identified differential metabolites were LPCs, which are regarded as second messengers involved in proinflammatory effects in both psoriasis and atherosclerosis (AS) [36, 37]. Monocytes stimulated by LPCs can produce IL-1β to further activate T lymphocytes to secrete IL-17A, which is an important pathogenic factor in psoriasis [38]. In addition, LPCs directly affect immunocytes, including monocytes, macrophages and T-lymphocytes [39–41]. Furthermore, IL-17A produced by stimulated T lymphocytes might also induce macrophage lipid uptake, which is a critical step in the pathophysiology of AS [42]. The cumulative literature emphasizes that high blood concentrations of LPCs are risk factors for psoriasis and CVDs. LPIs, which were another type of LPL identified in this study, exhibited a similar trend. LPIs are ligands of GPR55 and are detected in many immune organs/tissues, such as the spleen, thymus and blood immune cells [43]. Natural killer (NK) cells and monocytes activated by GPR55 can produce proinflammatory factors such as IL-12 and TNF-α, which have critical roles in the pathogeneses of psoriasis and CVDs [44]. Elevated LPIs levels have also been reported in a quantitative profiling study on high-fat diet-fed apolipoprotein E-deficient mice. Compared with other LPLs, LPIs continuously accumulate with worsening of AS and are considered stable biomarkers of AS [45]. Because activation of GPR55 by LPIs can cause intracellular overload and increase calcium release to accelerate vascular calcification, elevations in LPIs levels are regarded as triggers of AS [45].

The levels of DAs and acylcarnitines were significantly decreased in psoriasis patients with or without coronary heart disease in the two study cohorts. In a large-scale metabolic profiling study, acylcarnitines were considered an independent factor associated with the mortality of cardiovascular events [46]. Azelaic acid (AzA), a DA, has anti-inflammatory, antioxidative and antibacterial effects and has been used as a medication for the treatment of acne vulgaris [47]. It has also been reported that AzA has an anti-atherosclerotic effect. In one study on low-density lipoprotein receptor-knockout mice, dietary AzA supplementation significantly decreased atherosclerotic lesions formation [48].

In addition to being clinically employed in the treatment of psoriasis and arthritis, IL-17 mAbs have also been investigated for their effects on CVDs. A recent study demonstrated that endothelial function measured by flow-mediated dilation was improved after 52 weeks of treatment with IL-17 mAbs [49]. It has also been reported that one-year IL-17 mAb therapy reduces the size of the lipid-rich necrotic core, a high-risk coronary plaque feature, as assessed by coronary computed tomography angiography, providing evidence that systemic treatment of psoriasis with IL-17 mAbs may be beneficial for CVDs treatment [50]. As mentioned previously, after 12 weeks of treatment with ixekizumab, the blood levels of the most significantly altered lipid metabolites, such as LPCs, LPIs and DAs, in psoriasis patients were restored to levels comparable to those of the CON group. In addition to the factors reported in psoriasis patients, dysregulated metabolism of lipids, especially LPLs, is considered a critical pathogenic factor in the progression of CVDs [51]. The results of this study are consistent with the mentioned findings, indicating that IL-17A mAbs have the potential to reduce cardiovascular risk while ameliorating psoriatic lesions.

### Study strengths and limitations

This study had several strengths. First, although there have been many metabolomic analyses comparing healthy people and psoriasis patients, few studies have performed metabolic profiling of changes induced by IL-17A mAb treatment, eapecially alterations in lipid metabolism, in psoriasis patients. Second, to further explore whether IL-17A mAb therapy has a potential protective effect against CVDs, additional CON, PSO, PC and CV groups were included in study cohort 2. However, this study also had some notable limitations. First, the sample sizes of both study cohort 1 and study cohort 2 were small, which may have increased the risk of false-positive results. Second, lipid metabolism is related to dietary preferences and lifestyle habits; these factors were not taken into account in this study. Third, although most lipidic alterations were ameliorated after 12 weeks of ixekizumab treatment in the two study cohorts, more data are required to provide clearer evidence of how IL-17A mAbs regulates abnormal lipid metabolism, especially with regard to the role of Th17 cells. Nevertheless, this study on the lipid changed metabolites associated with IL-17A mAb treatment could provide a foundation for future in-depth research on the pathogenesis of psoriasis.

## Conclusion

This metabolomic analysis regarding lipid metabolism identified differential metabolites in psoriasis patients that were also significantly dysregulated in psoriasis patients with coronary heart disease. The study also revealed that the levels of most of these metabolites were restored after treatment with an IL-17A mAb. Since dysregulated lipid metabolism has been regarded as the critical factor in cardiovascular events, the recovery of lipid profiles in psoriasis patients indicates that IL-17A mAbs might have a protective effect against CVDs. Although IL-17A mAbs cannot be used to effectively treat cardiovascular events, further studies may provide novel therapies for psoriasis patients with CVDs or for cardiovascular patients.

## Supplementary Information

**Additional file 1: Table S1.** Differential metabolites between different groups. **Table S2.** ROC analysis results for potential biomarkers. **Fig. S1. a, b.** PCA score plots of the CON, PSO and IXE groups in positive ion and negative ion modes in study cohort 1. **c, d.** OPLS-DA score plots of the CON group versus the PSO group in positive and negative ion modes. **e, f.** Score plots of OPLS-DA model for the IXE group versus the PSO group in positive and negative ion modes. The CON group is indicated with green circles, the PSO group with red circles and the IXE group with blue circles. CON: group of healthy controls; PSO: group of psoriasis patients; IXE: group of ixekizumab-treated psoriasis patients. **Fig. S2.** Screening criteria for the differential metabolites identified in the IXE/PSO comparison in study cohort 1. **a.** Volcano plot showing the variations in metabolites in the IXE/PSO comparison according to the -log(*P*-value). **b.** S-plots for covariance and reliability correlations from OPLS-DA in the IXE/PSO comparison. **c.** Thirty-one identified differential metabolites in the IXE/PSO comparison. The bar plots represent, from left to right, the −log(*P*-value) outcomes from the t-test, the fold change and the VIP values obtained from OPLS-DA. PSO: group of psoriasis patients; IXE: group of ixekizumab-treated psoriasis patients. **Fig S3.** All the identified metabolites are conducted on ROC curve analysis, which is generally considered to be the gold standard for the assessment of biomarkers performance. **a.** The ROC curve of 37 differential metabolites and heatmap of area under the curve in the PSO/CON comparison. **b. **The ROC curve of 31 differential metabolites and heatmap of area under the curve in the IXE/PSO comparison. CON: group of healthy controls; PSO: group of psoriasis patients; IXE: group of ixekizumab-treated psoriasis patients.

## Data Availability

The datasets used or analyzed in this study are available from the corresponding author on reasonable request.

## Abbreviations

AzA: Azelaic acid
AUC: Area under the curve
CON: Group of healthy controls
CVDs: Cardiovascular diseases
CV: Group of coronary heart disease patients without psoriasis
DAs: Dicarboxylic acids
ESI: Electrospray ionization
FFAs: Free fatty acids
GPC: Glycerophosphocholine
IL-17A mAb: Interleukin-17A monoclonal antibody
IXE: Group of ixekizumab-treated psoriasis patients
LPLs: Lysophospholipids
LPAs: Lysophosphatidic acids
LPCs: Lysophosphatidylcholines
LPIs: Lysophosphatidylinositols
NK cells: Natural killer cells
OPLS-DA: Orthogonal partial least squares discrimination analysis
PCA: Principal component analysis
PC: Psoriasis patients with coronary heart diseases group
PSO: Group of psoriasis patients
QCs: Quality control samples
ROC: Receiver operating characteristic
sPLS-DA: Sparse partial least squares discriminant analysis
UPLC-MS: Ultra-performance liquid chromatography mass spectrometry
VIP: Variable importance in projection
