# Supplementary material for: Metabolic profiling reveals interleukin-17A monoclonal antibody treatment ameliorate lipids metabolism with the potentiality to reduce cardiovascular risk in psoriasis patients
Source: Lipids Health Dis. 2021 Feb 18;20:16. doi: 10.1186/s12944-021-01441-9 (PMC7890626; doi:10.1186/s12944-021-01441-9)

**SUPPLEMENTARY INFORMATON**

Metabolic profiling reveals interleukin-17A monoclonal antibody treatment ameliorate lipids metabolism with the potentiality to reduce cardiovascular risk in psoriasis patients

Han Cao^1,†^, Shengmin Su^1,†^, Qi Yang^2^, Yunchen Le^2^, Lihong Chen^2^, Mengyan Hu^2^, Xiaoyu Guo^1^, Jie Zheng^2^, Xia Li^2*^, Yunqiu Yu^1*^

^1^School of Pharmacy, Fudan University, Shanghai 201203, PR China

^2^Department of Dermatology, Ruijin Hospital, School of Medicine, Shanghai Jiaotong University, Shanghai 200025, PR China

^†^These authors contributed equally

^*^Corresponding author: Yunqiu Yu, Email: yqyu@shmu.edu.cn; Xia Li, Email: drlixia@126.com

**Table S1.** Differential metabolites between different groups

| Metabolites | RT  (min) | *m/z* | level | *P-*value | | VIP value | | | Fold change value | | | |
| --- | --- | --- | --- | --- | --- | --- | --- | --- | --- | --- | --- | --- |
|  |  |  |  | CON/PSO | IXE/PSO | | CON/PSO | IXE/PSO | CON/PSO | Trend | IXE/PSO | Trend |
| LPC (16:1) | 11.15 | 494.3250 | 2 | 0.008 | 0.001 | | 0.27 | 1.83 | 1.38 | ↑ | 0.77 | ↓ |
| LPC (18:0) | 14.63 | 546.3530 | 1 | 5.65E-05 | 1.06E-05 | | 1.94 | 6.06 | 1.44 | ↑ | 0.62 | ↓ |
| LPC (18:1) | 12.86 | 522.3559 | 1 | 0.001 | 4.16E-07 | | 5.34 | 10.95 | 1.33 | ↑ | 0.60 | ↓ |
| LPC (18:2) | 11.69 | 520.3403 | 1 | 1.12E-09 | 4.78E-10 | | 18.69 | 22.92 | 1.56 | ↑ | 0.56 | ↓ |
| LPC (20:2) | 13.45 | 592.3610 | 2 | 1.12E-09 | 0.009 | | 2.61 | 1.78 | 1.93 | ↑ | 0.83 | ↓ |
| LPC (20:3) | 12.31 | 546.3558 | 2 | 2.09E-08 | 8.72E-08 | | 8.8 | 8.80 | 3.26 | ↑ | 0.37 | ↓ |
| LPC (20:4) | 11.76 | 544.3400 | 2 | 1.02E-08 | 3.76E-05 | | 12.62 | 13.35 | 1.96 | ↑ | 0.46 | ↓ |
| LPC (20:5) | 11.69 | 542.3222 | 2 | 1.13E-09 | 2.64E-10 | | 7.67 | 9.42 | 1.39 | ↑ | 0.64 | ↓ |
| LPC (22:5) | 12.14 | 570.3556 | 2 | 4.64E-12 | 5.51E-08 | | 3.67 | 3.65 | 3.30 | ↑ | 0.30 | ↓ |
| LPC (22:6) | 11.73 | 568.3402 | 2 | 1.09E-07 | 9.46E-10 | | 6.27 | 7.73 | 1.88 | ↑ | 0.44 | ↓ |
| LPC (O-16:0) | 12.74 | 482.3609 | 2 | 0.382 | 5.85E-05 | | 0.41 | 2.23 | 1.08 | ↑ | 0.67 | ↓ |
| LPC (O-18:0) | 15.38 | 510.3919 | 2 | 0.203 | 8.69E-07 | | 0.42 | 1.41 | 1.12 | ↑ | 0.59 | ↓ |
| LPE (18:0) | 14.53 | 482.3245 | 2 | 0.005 | 0.001 | | 1.62 | 1.98 | 1.43 | ↑ | 0.72 | ↓ |
| LPI (16:0) | 12.34 | 571.2903 | 1 | 3.49E-13 | 0.001 | | 2.87 | 2.23 | 3.65 | ↑ | 0.65 | ↓ |
| LPI (18:0) | 15.25 | 599.3199 | 1 | 9.98E-14 | 0.001 | | 6.77 | 4.46 | 2.85 | ↑ | 0.75 | ↓ |
| LPI (18:2) | 11.66 | 595.2893 | 2 | 2.98E-10 | 0.012 | | 3.59 | 2.23 | 2.01 | ↑ | 0.83 | ↓ |
| LPI (20:4) | 11.77 | 619.2886 | 1 | 3.39E-14 | 0.001 | | 5.97 | 4.27 | 2.54 | ↑ | 0.76 | ↓ |
| LPA (20:4) | 13.64 | 457.2363 | 1 | 0.002 | 0.043 | | 2.38 | 1.16 | 2.54 | ↑ | 0.87 | ↓ |
| S1P | 10.58 | 378.2414 | 1 | 9.64E-08 | 0.005 | | 2.15 | 1.72 | 1.45 | ↑ | 0.85 | ↓ |
| PC (35:6) | 18.94 | 781.5571 | 2 | 0.019 | 0.026 | | 3.51 | 3.31 | 0.75 | ↓ | 1.37 | ↑ |
| PC (20:4/18:0) | 18.94 | 810.6010 | 2 | 0.154 | 0.026 | | 3.31 | 4.59 | 0.62 | ↓ | 1.90 | ↑ |
| L-Carnitine | 1.03 | 162.1123 | 1 | 0.003 | 0.008 | | 2.58 | 2.29 | 1.17 | ↑ | 0.87 | ↓ |
| L-Acetylcarnitine | 1.04 | 204.1226 | 2 | 1.48E-14 | 1.41E-08 | | 4.13 | 3.36 | 0.43 | ↓ | 2.01 | ↑ |
| L-Octanoylcarnitine | 6.42 | 288.2172 | 1 | 0.001 | 0.877 | | 1.43 | 0.97 | 0.60 | ↓ | 0.97 | ↓ |
| 9-Decenoylcarnitine | 7.19 | 314.2328 | 2 | 4.82E-05 | 0.731 | | 1.68 | 0.94 | 0.58 | ↓ | 0.94 | ↓ |
| Decanoylcarnitine | 7.86 | 316.2486 | 2 | 0.002 | 0.944 | | 1.88 | 1.01 | 0.59 | ↓ | 1.01 | ↑ |
| Glycerophosphocholine | 0.95 | 280.0921 | 1 | 7.14E-23 | 4.05E-12 | | 3.55 | 1.61 | 6.04 | ↑ | 0.17 | ↓ |
| Palmitic amide | 17.91 | 256.2637 | 1 | 0.005 | 0.008182218 | | 2.09 | 5.71 | 1.87 | ↑ | 0.56 | ↓ |
| Oleamide | 18.62 | 282.2796 | 1 | 0.002 | 0.008 | | 5.56 | 2.11 | 1.85 | ↑ | 0.55 | ↓ |
| Palmitic acid | 17.91 | 256.2637 | 1 | 4.83E-06 | 0.524 | | 1.34 | 0.96 | 1.64 | ↑ | 0.21 | ↓ |
| Oleic acid | 20.29 | 281.2479 | 1 | 2.27E-06 | 0.394 | | 2.01 | 0.93 | 1.58 | ↑ | 0.64 | ↓ |
| Linoleic acid | 18.97 | 279.2325 | 1 | 2.27E-06 | 0.280 | | 2.75 | 0.94 | 1.63 | ↑ | 0.47 | ↓ |
| Arachidonic acid | 18.74 | 303.2327 | 1 | 0.001 | 0.752 | | 1.35 | 0.90 | 1.26 | ↑ | 0.17 | ↓ |
| 13-Hydroxyhexadecanoic acid | 17.01 | 271.2277 | 2 | 0.010 | 0.031 | | 2.68 | 2.28 | 1.70 | ↑ | 0.94 | ↓ |
| 3-Hydroxyvaleric acid | 4.06 | 117.0553 | 2 | 0.002 | 0.086 | | 1.91 | 0.83 | 1.70 | ↑ | 0.91 | ↓ |
| 3-Methyl-2-oxovaleric acid | 4.82 | 129.0552 | 2 | 1.75E-05 | 0.151 | | 1.67 | 0.90 | 1.35 | ↑ | 0.86 | ↓ |
| 3-Hydroxydodecanedioic acid | 6.35 | 245.1384 | 2 | 4.05E-06 | 0.009 | | 4.48 | 8.36 | 1.31 | ↑ | 1.31 | ↑ |
| Suberic acid | 5.24 | 173.0828 | 2 | 0.019 | 0.002 | | 1.56 | 1.54 | 0.39 | ↓ | 2.48 | ↑ |
| Azelaic acid | 5.83 | 187.0965 | 1 | 0.013 | 0.056 | | 8.88 | 2.09 | 0.46 | ↓ | 11.4 | ↑ |
| Sebacic acid | 6.41 | 201.1125 | 2 | 0.019 | 0.002 | | 2.09 | 2.28 | 0.47 | ↓ | 2.35 | ↑ |
| Undecanedioic acid | 7.08 | 215.1284 | 1 | 1.63E-05 | 0.013 | | 2.77 | 3.94 | 0.43 | ↓ | 2.47 | ↑ |
| 9-Oxohexadecanoic acid | 11.31 | 329.2334 | 2 | 0.002 | 0.006 | | 0.63 | 1.51 | 0.75 | ↓ | 1.96 | ↑ |
| Indoxyl sulfate | 4.65 | 212.0021 | 2 | 0.001 | 0.624 | | 2.12 | 0.65 | 2.48 | ↑ | 0.85 | ↓ |

CON: group of healthy controls; PSO: group of psoriasis patients; IXE: group of ixekizumab-treated psoriasis patients; RT: retention time.

**Table S2.** ROC analysis results for potential biomarkers

| Metabolites | RT (min) | *m/z* | AUC | |
| --- | --- | --- | --- | --- |
|  |  |  | PSO/CON | IXE/PSO |
| LPC (16:1) | 11.15 | 494.3250 | NA | 0.67 |
| LPC (18:0) | 14.63 | 546.3530 | 0.78 | 0.84 |
| LPC (18:1) | 12.86 | 522.3559 | 0.76 | 0.87 |
| LPC (18:2) | 11.69 | 520.3403 | 0.93 | 0.95 |
| LPC (20:2) | 13.45 | 592.3610 | 0.88 | 0.64 |
| LPC (20:3) | 12.31 | 546.3558 | 0.94 | 0.91 |
| LPC (20:4) | 11.76 | 544.3400 | 0.91 | 0.91 |
| LPC (20:5) | 11.69 | 542.3222 | 0.93 | 0.94 |
| LPC (22:5) | 12.14 | 570.3556 | 0.95 | 0.94 |
| LPC (22:6) | 11.73 | 568.3402 | 0.88 | 0.91 |
| LPC (O-16:0) | 12.74 | 482.3609 | NA | 0.81 |
| LPC (O-18:0) | 15.38 | 510.3919 | NA | 0.88 |
| LPE (18:0) | 14.53 | 482.3245 | 0.73 | 0.71 |
| LPI (16:0) | 12.34 | 571.2903 | 0.96 | 0.74 |
| LPI (18:0) | 15.25 | 599.3199 | 0.97 | 0.68 |
| LPI (18:2) | 11.66 | 595.2893 | 0.93 | 0.65 |
| LPI (20:4) | 11.77 | 619.2886 | 0.97 | 0.70 |
| LPA (20:4) | 13.64 | 457.2363 | 0.74 | 0.6 |
| S1P | 10.58 | 378.2414 | 0.87 | 0.68 |
| PC (35:6) | 18.94 | 781.5571 | 0.69 | 0.73 |
| PC (20:4/18:0) | 18.94 | 810.6010 | NA | 0.70 |
| L-Carnitine | 1.03 | 162.1123 | 0.73 | 0.69 |
| L-Acetylcarnitine | 1.04 | 204.1226 | 0.98 | 0.93 |
| L-Octanoylcarnitine | 6.42 | 288.2172 | 0.77 | NA |
| 9-Decenoylcarnitine | 7.19 | 314.2328 | 0.82 | NA |
| Decanoylcarnitine | 7.86 | 316.2486 | 0.75 | NA |
| Glycerophosphocholine | 0.95 | 280.0921 | 1 | 0.99 |
| Palmitic amide | 17.91 | 256.2637 | 0.70 | 0.69 |
| Oleamide | 18.62 | 282.2796 | 0.73 | 0.72 |
| Palmitic acid | 17.91 | 256.2637 | 0.86 | NA |
| Oleic acid | 20.29 | 281.2479 | 0.86 | NA |
| Linoleic acid | 18.97 | 279.2325 | 0.80 | NA |
| Arachidonic acid | 18.74 | 303.2327 | 0.73 | NA |
| 13-Hydroxyhexadecanoic acid | 17.01 | 271.2277 | 0.88 | 0.77 |
| 3-Hydroxyvaleric acid | 4.06 | 117.0553 | 0.83 | 0.61 |
| 3-Methyl-2-oxovaleric acid | 4.82 | 129.0552 | 0.84 | NA |
| 3-Hydroxydodecanedioic acid | 6.35 | 245.1384 | 0.71 | NA |
| Suberic acid | 5.24 | 173.0828 | 0.85 | 0.67 |
| Azelaic acid | 5.83 | 187.0965 | 0.85 | NA |
| Sebacic acid | 6.41 | 201.1125 | 0.84 | 0.67 |
| Undecanedioic acid | 7.08 | 215.1284 | 0.85 | 0.67 |
| 9-Oxohexadecanoic acid | 11.31 | 329.2334 | NA | 0.67 |
| Indoxyl sulfate | 4.65 | 212.0021 | 0.83 | NA |

CON: group of healthy controls; PSO: group of psoriasis patients; IXE: group of ixekizumab-treated psoriasis patients; AUC: area under the curve; RT: retention time.

**Fig. S1.** **a, b.** PCA score plots of the CON, PSO and IXE groups in positive ion and negative ion modes in study cohort 1. **c, d.** OPLS-DA score plots of the CON group versus the PSO group in positive and negative ion modes. **e, f.** Score plots of OPLS-DA model for the IXE group versus the PSO group in positive and negative ion modes. The CON group is indicated with green circles, the PSO group with red circles and the IXE group with blue circles. CON: group of healthy controls; PSO: group of psoriasis patients; IXE: group of ixekizumab-treated psoriasis patients.


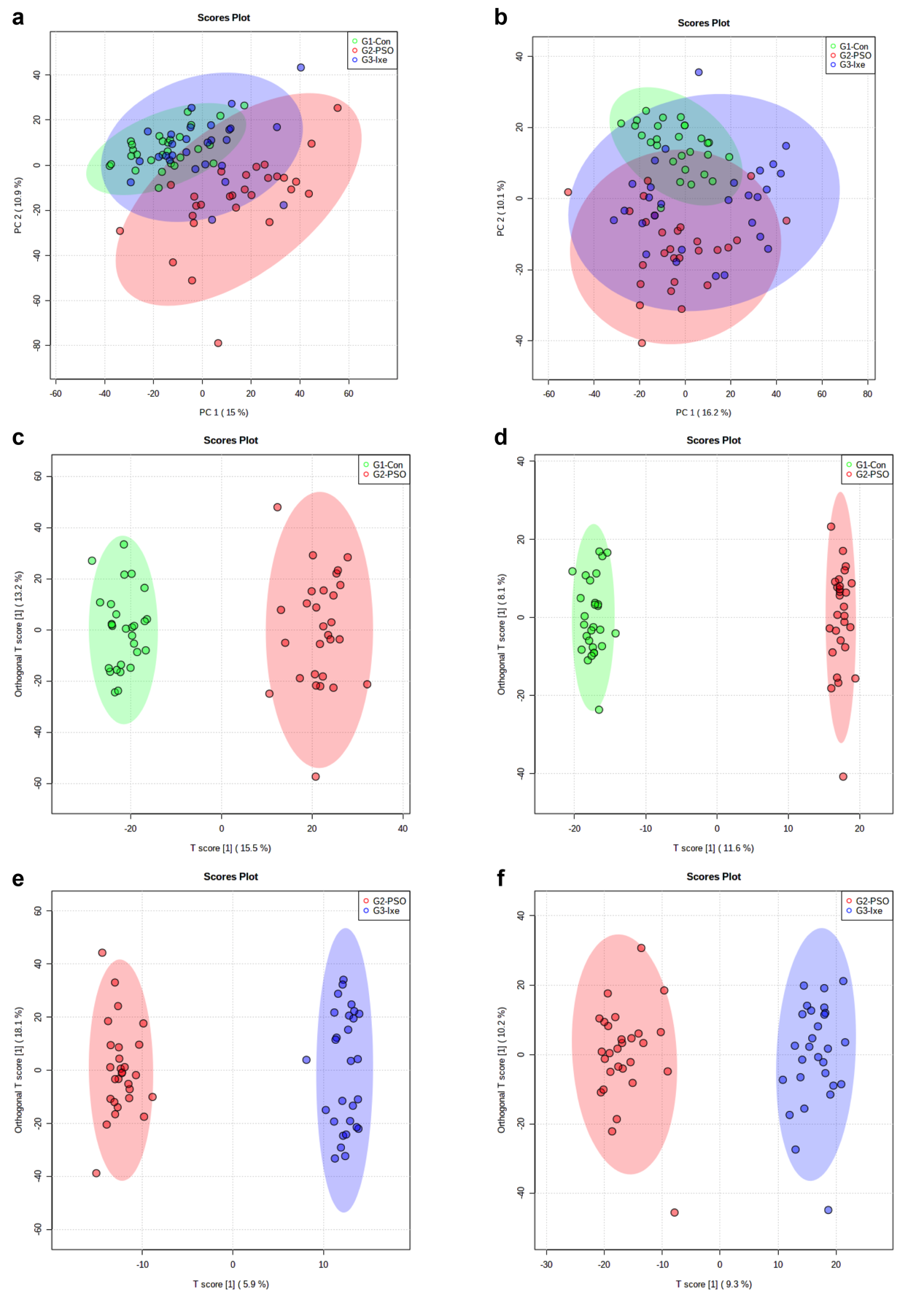


**Fig. S2.** Screening criteria for the differential metabolites identified in the IXE/PSO comparison in study cohort 1. **a.** Volcano plot showing the variations in metabolites in the IXE/PSO comparison according to the -log(*P*-value). **b.** S-plots for covariance and reliability correlations from OPLS-DA in the IXE/PSO comparison. **c.** Thirty-one identified differential metabolites in the IXE/PSO comparison. The bar plots represent, from left to right, the −log(*P*-value) outcomes from the t-test, the fold change and the VIP values obtained from OPLS-DA. PSO: group of psoriasis patients; IXE: group of ixekizumab-treated psoriasis patients.


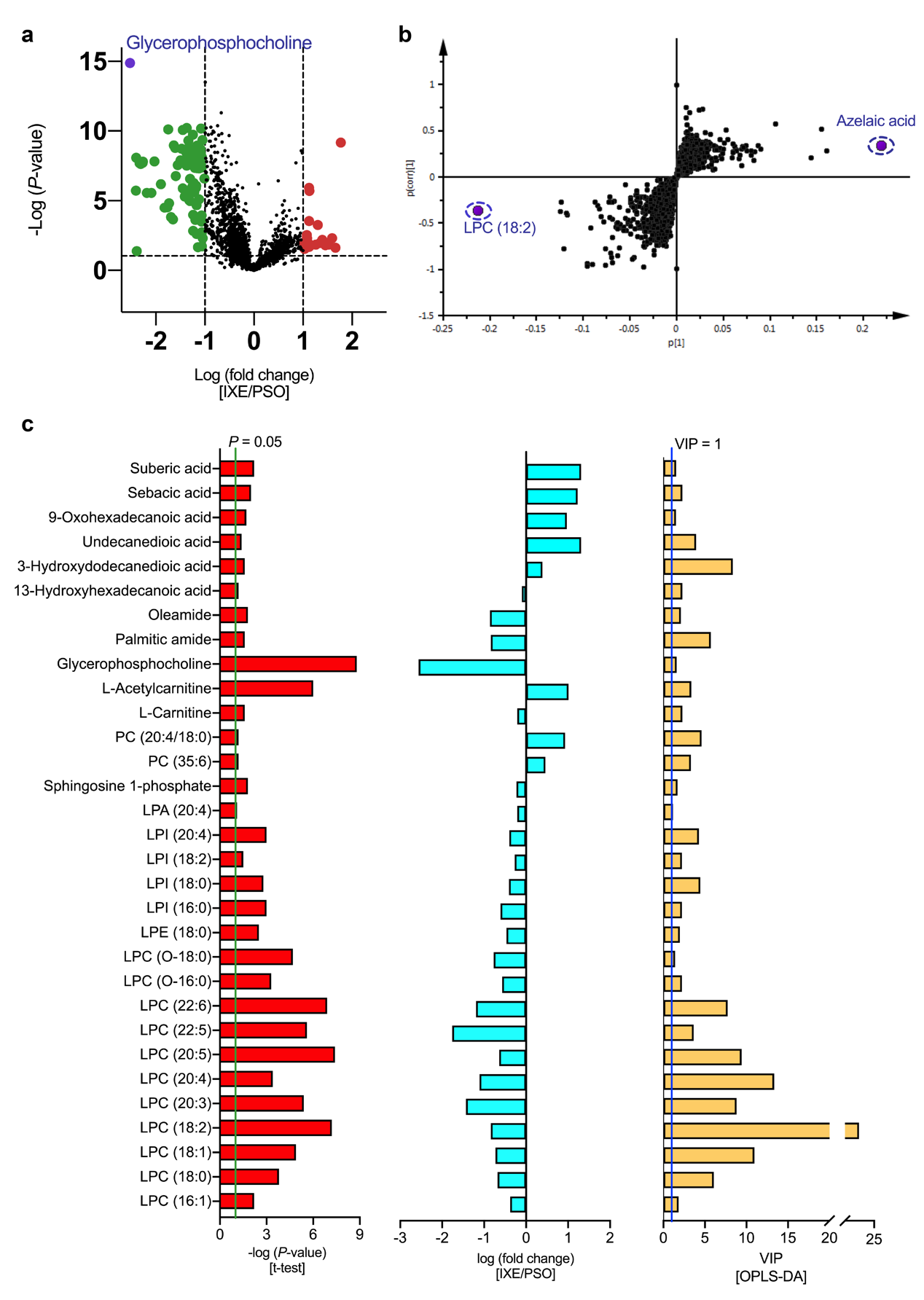


**Fig S3.** All the identified metabolites are conducted on ROC curve analysis, which is generally considered to be the gold standard for the assessment of biomarkers performance. **a.** The ROC curve of 37 differential metabolites and heatmap of area under the curve in the PSO/CON comparison. (b) The ROC curve of 31 differential metabolites and heatmap of area under the curve in the IXE/PSO comparison. CON: group of healthy controls; PSO: group of psoriasis patients; IXE: group of ixekizumab-treated psoriasis patients.


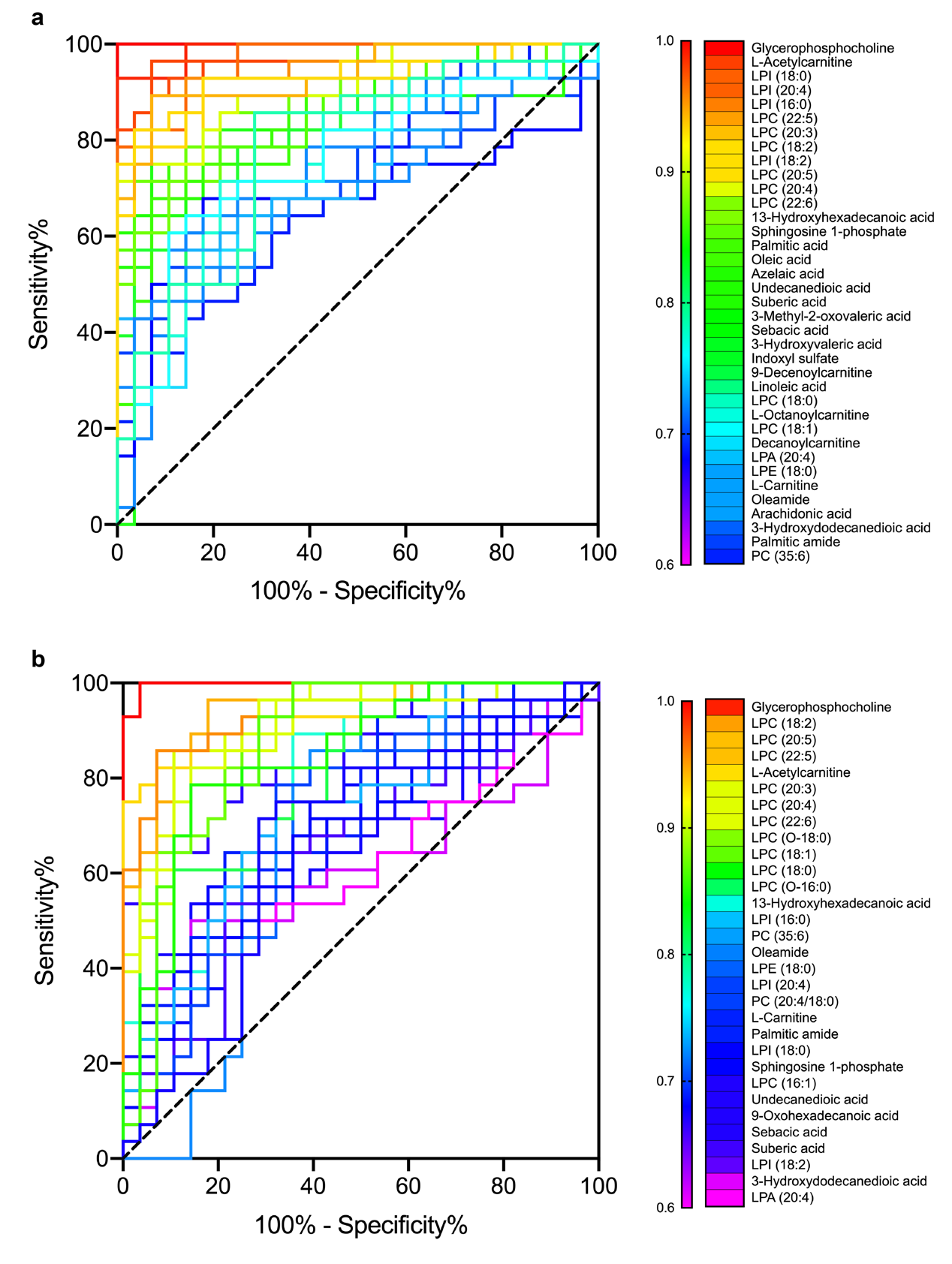

Supplement: Supplementary file 1 — Additional file 1: Table S1. Differential metabolites between different groups. Table S2. ROC analysis results for potential biomarkers. Fig. S1. a, b. PCA score plots of the CON, PSO and IXE groups in positive ion and negative ion modes in study cohort 1. c, d. OPLS-DA score plots of the CON group versus the PSO group in positive and negative ion modes. e, f. Score plots of OPLS-DA model for the IXE group versus the PSO group in positive and negative ion modes. The CON group is indicated with green circles, the PSO group with red circles and the IXE group with blue circles. CON: group of healthy controls; PSO: group of psoriasis patients; IXE: group of ixekizumab-treated psoriasis patients. Fig. S2. Screening criteria for the differential metabolites identified in the IXE/PSO comparison in study cohort 1. a. Volcano plot showing the variations in metabolites in the IXE/PSO comparison according to the -log(P-value). b. S-plots for covariance and reliability correlations from OPLS-DA in the IXE/PSO comparison. c. Thirty-one identified differential metabolites in the IXE/PSO comparison. The bar plots represent, from left to right, the −log(P-value) outcomes from the t-test, the fold change and the VIP values obtained from OPLS-DA. PSO: group of psoriasis patients; IXE: group of ixekizumab-treated psoriasis patients. Fig S3. All the identified metabolites are conducted on ROC curve analysis, which is generally considered to be the gold standard for the assessment of biomarkers performance. a. The ROC curve of 37 differential metabolites and heatmap of area under the curve in the PSO/CON comparison. b. The ROC curve of 31 differential metabolites and heatmap of area under the curve in the IXE/PSO comparison. CON: group of healthy controls; PSO: group of psoriasis patients; IXE: group of ixekizumab-treated psoriasis patients. [file 12944_2021_1441_MOESM1_ESM.docx]
